# Evaluating the causal effect of circulating proteome on the risk of inflammatory bowel disease-related traits using Mendelian randomization

**DOI:** 10.3389/fimmu.2024.1434369

**Published:** 2024-07-31

**Authors:** Beining Li, Ping Hu, Hongyan Liang, Xingliang Zhao, Aiting Zhang, Yingchong Xu, Bin Zhang, Jie Zhang

**Affiliations:** ^1^ Department of Gastroenterology and Hepatology, Tianjin Medical University General Hospital, Tianjin Institute of Digestive Diseases, Tianjin Key Laboratory of Digestive Diseases, Tianjin, China; ^2^ The Second Hospital of Tianjin Medical University, Tianjin, China; ^3^ Department of Orthopedic, Tianjin Medical University General Hospital, Tianjin, China

**Keywords:** inflammatory bowel disease, mendelian randomization, protein, therapeutic target, singer cell

## Abstract

**Objective:**

This study sought to identify circulating proteins causally linked to Inflammatory Bowel Disease (IBD) traits through a Mendelian Randomization (MR) analytical framework.

**Methods:**

Using a large-scale, two-sample MR approach, we estimated the genetic links of numerous plasma proteins with IBD and its subtypes, leveraging information from the Inflammatory Bowel Disease Genetics Consortium. To assess the robustness of MR findings, methods like Bayesian colocalization, and Steiger filtering analysis, evaluation of protein-altering variants. Further insights into IBD’s underlying mechanisms and therapeutic targets were gleaned from single-cell sequencing analyses, protein-protein interaction assessments, pathway enrichment analyses, and evaluation of drug targets.

**Results:**

By cis-only MR analysis, we identified 83 protein-phenotype associations involving 27 different proteins associated with at least one IBD subtype. Among these proteins, DAG1, IL10, IL12B, IL23R, MST1, STAT3 and TNFRSF6B showed overlapping positive or negative associations in all IBD phenotypes. Extending to cis + trans MR analysis, we further identified 117 protein-feature associations, including 44 unique proteins, most of which were not detected in the cis-only analysis. In addition, by performing co-localization analysis and Steiger filtering analysis on the prioritized associations, we further confirmed the causal relationship between these proteins and the IBD phenotype and verified the exact causal direction from the protein to the IBD-related feature.

**Conclusion:**

MR analysis facilitated the identification of numerous circulating proteins associated with IBD traits, unveiling protein-mediated mechanisms and promising therapeutic targets.

## 1 Introduction

Inflammatory Bowel Disease (IBD), comprising Crohn’s Disease (CD) and Ulcerative Colitis (UC), is marked by idiopathic, non-specific inflammatory intestinal disorders. IBD is characterized by a chronic, intractable course, necessitating extended periods of treatment and attentive care (1). The combination of endoscopy and histological analysis is universally acknowledged as the gold standard for diagnosing and assessing IBD. However, it is costly and invasive, potentially causing discomfort and pain for the patient (2). C-reactive protein (CRP) is a viable serum biomarker for monitoring IBD treatment. Although CRP is effective, it is not exclusively specific for IBD because any inflammation can lead to elevated CRP levels (3). Anti-inflammatory medicines and biologics are currently used to treat IBD. Anti-inflammatory medicines, such as 5-Aminosalicylates (5-ASA) and corticosteroids, are the initial step in IBD treatment (4), whereas biologics are mainly employed when there is no improvement after 5-ASA and steroid treatment (5). Nevertheless, some patients are insensitive to biologics. Hence, it is imperative to explore more efficient and non-invasive biomarkers for monitoring IBD treatment, alongside implementing more effective treatment approaches to reduce the physical and financial strain on individuals, families, and society.

Circulating plasma proteins, as key regulatory factors in most molecular pathways within the organism, hold immense potential in disease diagnosis and treatment. Earlier research indicates the potential involvement of circulating plasma proteins in the development and advancement of IBD, along with their potential therapeutic benefits. A case-control study involving 98 UC patients and 105 CD patients revealed the involvement of immune-mediated interleukin (IL)-22 in the development of IBD (6). A prospective study revealed that α-2 macroglobulin (LRG), interleukin (IL)-6, prealbumin (pre-Alb), high-sensitivity CRP (hs-CRP), CRP, and fecal calprotectin (FC) are associated with changes in UC disease activity, with LRG also reflecting disease activity during anti-tumor necrosis factor (TNF) antibody treatment (7). Genomics and proteomics are essential scientific research tools for studying the composition, structure, function, interactions, and regulatory mechanisms of genes and proteins within organisms. Genomic research provides foundational data for proteomics, while proteomics offers more comprehensive bioinformatics data for genomics. The amalgamation of genomic and proteomic data enables the examination of genetic differences linked to protein expression levels, also known as protein quantitative trait loci (pQTL). pQTL research can help understand the relationship between genotype and protein expression, revealing the regulatory mechanisms of genotype on protein expression, and providing a comprehensive and crucial information base for understanding the mechanisms of disease onset and progression, prevention, and precision treatment (8).

Mendelian Randomization (MR) functions as a statistical method in epidemiological studies to evaluate the causal association between exposure factors and outcomes, employing genetic variations highly correlated with the exposure factors as instrumental variables (9). By combining MR and pQTL, the causal relationship between specific genotypes and protein quantities can be evaluated, allowing for the more accurate identification of pQTL affecting protein quantities. For example, in a recent two-sample MR analysis utilizing pQTL data from seven large-scale proteomic studies, 13 protein biomarkers linked to a higher risk of colorectal cancer were identified (10). Another two-sample MR combined with colocalization analysis utilizing data on 4907 levels of circulating plasma proteins, identified links between these proteins and IBD and its subtypes. The amalgamation of drug databases data led to the deduction that circulating plasma proteins MST1, HGFAC, STAT3, ITPKA, and CXCL5 could be viable drug targets for IBD or UC (11). The objective of this study is to identify circulating plasma proteins associated with IBD and its subtypes (CD and UC) through MR analysis, delving into protein-mediated mechanisms and potential therapeutic targets.

**Figure 1:**
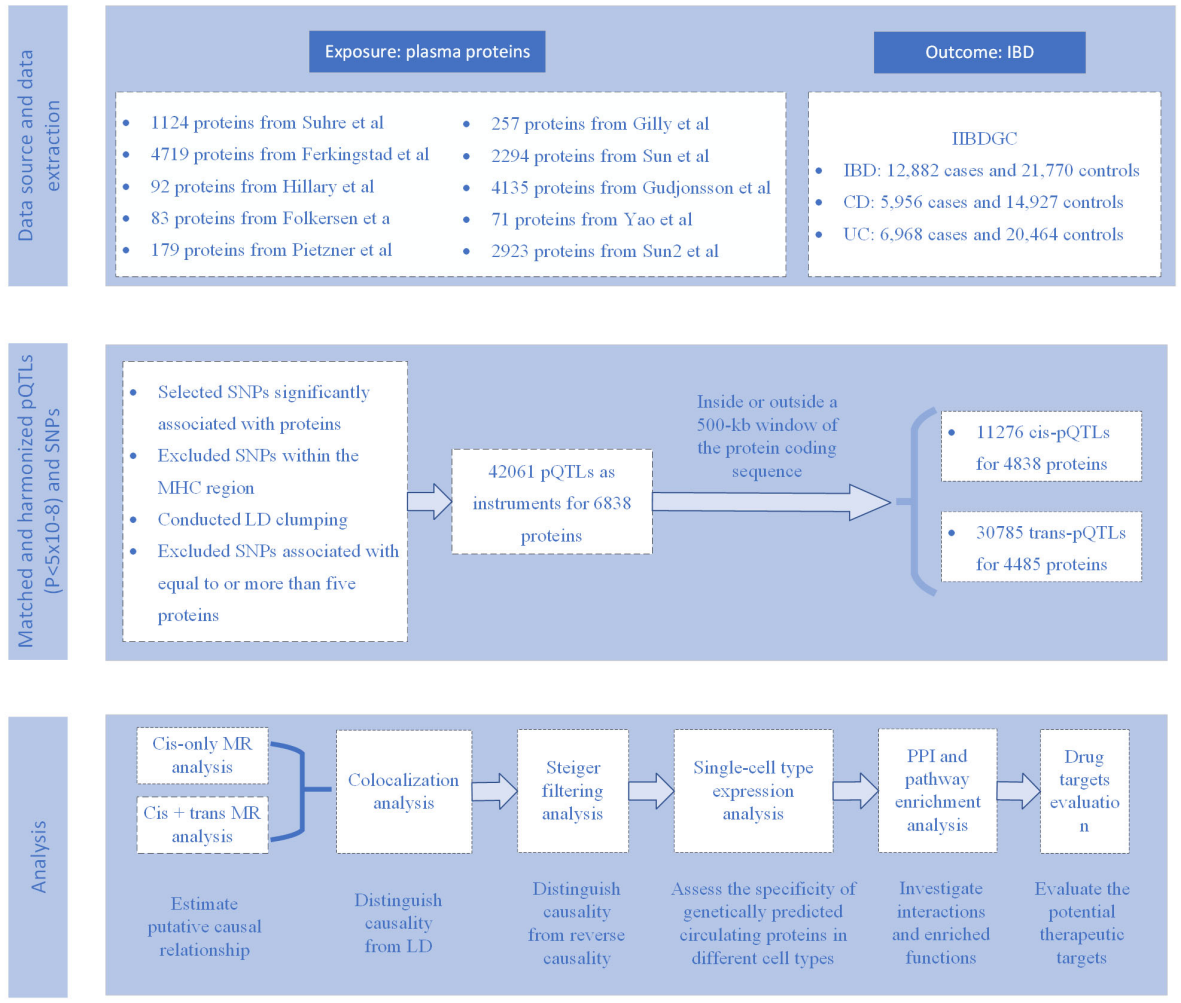
Flow chart.

This study (**Figure 1**) aims to explore the association between genetically predicted circulating proteins and IBD and its subtypes. Using the MR method, we constructed MR tools based on 10 proteomic GWAS datasets, focusing on evaluating cis- and trans-acting serum pQTLs related to circulating proteins. Through a comprehensive statistical analysis of the data provided by IIBDGC on IBD, which includes MR analysis, Steiger filtering, and colocalization analysis, we have effectively identified the causal relationship between circulating proteins and IBD. Additionally, for proteins identified through MR analysis, we further evaluated their potential for drug targeting in pharmaceutical development. This comprehensive approach provides new perspectives on the involvement of circulating proteins in the pathogenesis of IBD and highlights potential targets for therapy, establishing a solid foundation for future treatment strategies for IBD.

## 2 Method and material

### 2.1 Exposure

The exposure factors in this study are genetically predicted circulating proteins, for which we utilized nine different cohort pQTL (protein Quantitative Trait Loci) data provided by Zhang’s study (12). Additionally, we supplemented this with the latest pQTL data (13) based on their selection criteria, enriching the research value. The MR tools for circulating proteins were constructed from ten proteomics GWAS (Genome-Wide Association Studies) (13–22). The details of these ten studies are presented in **Supplementary Table 1**. The selection of candidate instrumental variables, serum pQTLs, was conducted as follows: initially, single nucleotide polymorphisms (SNPs) associated with any protein were chosen based on the recommended p-value thresholds in the respective studies (**Supplementary Table 2**). Following this, SNPs situated in the major histocompatibility complex (MHC) region (chr6: 26 Mb to 34 Mb), recognized for its intricate linkage disequilibrium (LD) formations, were omitted. The third step involved LD clumping, where a threshold of r2 > 0.01 and a distance of <5000kb upstream/downstream were applied to identify independent pQTLs for each protein. To further ensure the robustness of our MR analysis, we specifically excluded weak instrumental variables, i.e., those with F-statistic values less than or equal to 10. Ultimately, instruments linked to five or more proteins were omitted owing to their significant pleiotropy. In this study, tools were categorized into cis-pQTLs, situated within a 500kb segment of the protein-coding sequence, and trans-pQTLs, positioned beyond the 500kb segment of the protein-coding gene.

### 2.2 Outcomes

To delve into the genetic underpinnings of IBD and its subtypes, CD and UC, the study employed aggregated data from the International IBD Genetics Consortium (IIBDGC, https://www.ibdgenetics.org/). The International IBD Genetics Consortium dataset comprises 12,882 IBD cases, including 5,956 CD cases and 6,968 UC cases (23), all of European ancestry to maintain genetic homogeneity (**Table 1**). These cases were diagnosed according to strict clinical criteria, encompassing radiological, endoscopic, and histopathological assessments. Genetic association analyses were adjusted for age, sex, and up to 20 principal genetic components to control for potential confounding factors, ensuring the accuracy and reliability of the analysis results and providing in-depth insights into the genetic characteristics of IBD and its subtypes.

**Table 1:** Disease data sources.

Phenotypes	Data source	Phenotypic code	Cases/Controls	Ancestry
IBD	Liu,et al (22)	ieu-a-31	12882/21770 = 34652	European
CD	Liu,et al (22)	ieu-a-30	5956/14927 = 20883	European
UC	Liu,et al (22)	ieu-a-32	6968/20464 = 27432	European

IBD, Inflammatory bowel disease.

CD, Crohn’s disease.

UC, Ulcerative colitis.

### 2.3 MR analysis

In the MR analysis, genetically predicted proteins were considered the exposure factor, with IBD and its subtypes (CD and UC) as the outcomes. MR analysis tools were constructed utilizing both cis-pQTLs and all pQTLs (cis + trans). The MR effect was assessed utilizing the Wald ratio estimator for a single pQTL, and the inverse variance weighted method for two or more available pQTLs. Given that our selection strategy might provide instrumental variables linked with up to four proteins, which could display pleiotropy, proteins-traits associations supported by MR evidence were scrutinized for their instruments if related with more than one protein. MR analyses were conducted, potential pleiotropic instruments were excluded, and results were presented. Additional sensitivity assessments were conducted to examine the solidity of the results and to tackle possible diversity and lateral pleiotropy across the instruments. In cases where Cochran’s Q test revealed variability across various instruments, the weighted median technique was utilized, permitting as much as 50% of the instruments to be invalid. Likewise, the MR-Egger technique was utilized in cases where horizontal pleiotropy was identified via the MR-Egger method, given its ability to adjust for instrument pleiotropy. The ‘TwoSampleMR’ R package (http://github.com/MRCIEU/TwoSampleMR) was utilized for MR analysis. For MR analysis, the threshold for the Bonferroni-adjusted p-value was established at 0.05, divided by the total proteins examined.

### 2.4 Steiger filtering analysis

The Steiger filtering technique, implemented via the ‘TwoSampleMR’ R package, was employed on MR associations passing the multiplicity-adjusted threshold to examine for potential distortions from reverse causation. For simpler understanding, results were categorized: ‘true’ when the exposure’s impact on the outcome was notable at p<0.05; ‘false’ when it was contrary at p<0.05; and ‘uncertain’ when p was 0.05 or more.

### 2.5 Colocalization analysis

Results from the surviving MR analysis were assessed through Bayesian colocalization analysis to calculate the likelihood of a single variant at each site impacting both protein and IBD-related characteristics, as opposed to coincidental variant sharing due to LD association. The ‘coloc’ R package (http://cran.r-project.org/web/packages/coloc) was utilized to assess colocalization. The ‘coloc’ R package offered posterior probabilities for five theories about the presence of a common variant in two traits, with the fourth hypothesis suggesting a link between both traits and the identical genetic variant in that area. Hypothesis 4’s posterior probability exceeding 0.8 was identified as robust proof of colocalization.

### 2.6 Single-cell type expression analysis

To understand the specificity of genetically predicted circulating proteins across different cell types, we opted to use the scIBD platform (http://scibd.cn/) (24), a cutting-edge tool specifically designed for single-cell meta-analysis in IBD. scIBD aggregates approximately 1.14 million single-cell data from various developmental stages (including fetal, child, and adult) and tissues from multiple anatomical regions, such as blood, small intestine, and colon, derived from 12 different datasets, reflecting different disease states including healthy, inflamed UC, and inflamed CD. Utilizing the scIBD platform, we were able to intricately analyze the transcriptomic features of 9 major cell subtypes (e.g., myeloid cells, CD4 T cells, CD8 T cells, etc.) and 101 cell subtypes. This was particularly important for our study as it allowed us to explore the expression and regulation of circulating proteins at the single-cell level, revealing their specificity across different cell types. Additionally, the interactive visualization tools provided by scIBD enabled us to more precisely analyze the expression patterns and gene regulatory networks of a given gene set in each cell subset. This in-depth single-cell analysis allowed us to more accurately identify key circulating proteins related to the pathogenesis of IBD, thereby revealing their specific roles in disease progression.

### 2.7 Protein-protein interaction and functional enrichment analysis

For exploring the connections between proteins ranked by MR, a protein-protein interaction (PPI) network was developed utilizing the Search Tool for the Retrieval of Interacting Genes (STRING, version 11.5, https://string-db.org/). Furthermore, the ‘ClusterProfiler’ R package (https://bioconductor.org/packages/release/bioc/html/clusterProfiler.html) was utilized for analyzing Gene Ontology (GO) and Kyoto Encyclopedia of Genes and Genomes (KEGG) pathways, aiming to investigate the possible biological roles and pathways linked to these proteins. For the enrichment analysis, the selection parameters were set thus: a p-value less than 0.05 signified notable enrichment outcomes. Gene ID conversion was performed using the ‘org.Hs.eg.db’ package, while the clusterProfiler package facilitated GO functional enrichment analysis, encompassing Biological Process (BP), Cellular Component (CC), and Molecular Function (MF), as well as KEGG pathway enrichment analysis to decipher the biological pathways engaged by DEGs (Differentially Expressed Genes) (25).

### 2.8 Identification of druggable proteins

To assess the druggability of identified proteins, this study queried the DGIdb database (https://old.dgidb.org/) and previously researched lists of druggable genes. Proteins identified in DGIdb, along with their associated drug names and development information, were documented. DGIdb aggregates information from various public databases and publications, including DrugBank, PharmGKB, ChEMBL, Drug Target Commons, among others, and updates it using specialized knowledge and text mining methods. Presently, the database houses in excess of 10,000 genes and 15,000 medications, encompassing over 50,000 interactions between drugs and genes, spanning 43 possible categories of drugs. DGIdb, as a tool for research, is crucial for investigating interactions between drugs and genes, as well as the genome that can be targeted for drug development, especially in clinical sequencing and tailored medical practices, serving as key sources for disease management and pharmaceutical advancement.

## 3 Results

### 3.1 Calculating the impact of plasma proteins on IBD through the application of cis-only MR

**Figure 2:**
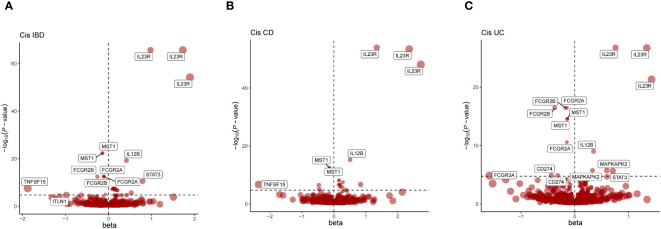
Presents a series of volcano plots from MR analyses, depicting the associations between genetically predicted levels of circulating proteins and IBD as well as its subtypes. Highlighted genes represent those that remained significant after Bonferroni correction in MR results (p<0.05/number of proteins tested). The x-axis beta values greater than zero indicate a positive influence of circulating proteins on the outcome, while values less than zero suggest a negative impact. **(A)** Volcano plot for IBD-related Cis-Only MR analysis; **(B)** Volcano plot for CD-related Cis-Only MR analysis; **(C)** Volcano plot for UC-related Cis-Only MR analysis.

Considering that cis-pQTLs are assumed to have a higher *a priori* likelihood of specific biological effects compared to trans-pQTLs, cis-pQTLs were initially used as genetic instruments for MR analysis to methodically evaluate the proof of causal effects of causative effects of plasma proteins on IBD and its subtypes (**Figure 2**).

In summary, using the Bonferroni adjustment threshold, with IBD p<0.05/4705, CD p<0.05/4704, UC p<0.05/4703 (**Supplementary Table 2**), 83 protein-phenotype association were identified, among which 27 distinct proteins were linked to at least one of the IBD subtypes (**Figure 2**, **Table 2**, **Supplementary Table 4**).

**Table 2:** Cis-pQTL-Based MR, Colocalization, and Steiger Filter Analysis: Final Protein Selections Associated with IBD and Its Subtypes.

Protein	Exposure	Outcome	Direction
CEBPB	Benjamin B. Sun2, et al	IBD	Positive
ERAP2	Alexander Gudjonsson, et al,Benjamin B. Sun, et al	IBD,CD	Positive
FCGR2A	Egil Ferkingstad , et al	UC,IBD	Negative
HGFAC	Alexander Gudjonsson, et al	IBD	Negative
IL10	Benjamin B. Sun2, et al	IBD,CD,UC	Negative
IL12B	Egil Ferkingstad , et al	IBD,CD,UC	Positive
IL18R1	Benjamin B. Sun, et al	IBD,CD	Positive
IL1RL1	Egil Ferkingstad , et al,Benjamin B. Sun, et al	IBD	Positive
IL23R	Karsten Suhre, et al,Benjamin B. Sun, et al,Alexander Gudjonsson, et al	IBD,CD,UC	Positive
MST1	Karsten Suhre, et al,Egil Ferkingstad , et al,	IBD,CD,UC	Negative
PARK7	Benjamin B. Sun2, et al	IBD	Negative
STAT3	Egil Ferkingstad , et al	CD	Positive
TNFRSF14	Benjamin B. Sun2, et al	IBD,UC	Negative
TNFRSF6B	Benjamin B. Sun2, et al	CD	Negative

Proteins like DAG1, IL10, IL12B, IL23R, MST1, STAT3, and TNFRSF6B showed overlapping positive associations across IBD, CD, and UC phenotypes (**Supplementary Table 5**), maintaining the same direction of association. For instance, the latest data from Benjamin B. Sun et al. indicate a negative correlation between genetically predicted IL10 and IBD, CD, and UC phenotypes (**Figure 2**, **Table 2**), suggesting a possible common pathogenesis mechanism among these phenotypes. Specifically, the beta coefficients of IL10 in IBD, CD and UC were -1.50822738, -1.258632705 and -1.670350144, respectively, suggesting that increased levels of IL10 are associated with a decreased risk of these disorders. DAG1 showed significant positive associations with IBD and its subtypes, with beta coefficients in IBD, CD and UC 2.72190224, 2.814379709, and 2.605902159, respectively. This suggests that an increase in DAG1 may be associated with an increased risk of IBD. The β coefficients of MST1 in IBD and its subtypes showed a negative correlation, which suggests that an increase in MST1 may be associated with a reduced risk of IBD. This provides clues to MST1 as a potential therapeutic target. Additionally, in the cis-pQTL MR sensitivity analysis, the newly identified ENTR1 protein was found to reduce the risk of IBD and CD incidence. The β coefficients of ENTR1 in CD and IBD were -0.519010561 and -0.331279952, respectively. This new finding emphasizes the potential role of ENTR1 in the pathogenesis of IBD (**Supplementary Table 6**).

### 3.2 Calculating the impact of plasma proteins on IBD through the application of cis + trans MR

**Figure 3:**
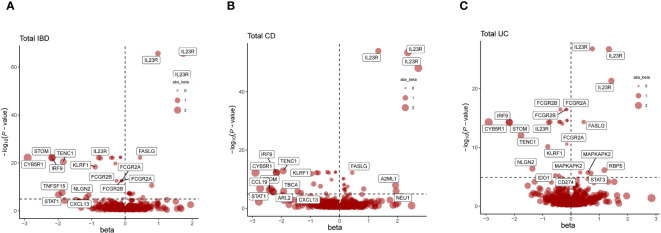
Presents a series of volcano plots from MR analyses, depicting the associations between genetically predicted levels of circulating proteins and IBD as well as its subtypes. Highlighted genes represent those that remained significant after Bonferroni correction in MR results (p<0.05/number of proteins tested). The x-axis beta values greater than zero indicate a positive influence of circulating proteins on the outcome, while values less than zero suggest a negative impact. **(A)** Volcano plot for the IBD-related cis + trans MR analysis. **(B)** Volcano plot for the CD-related cis + trans MR analysis. **(C)** Volcano plot for the UC-related cis + trans MR analysis.

Incorporating trans-pQTLs into the MR analysis potentially enhances the dependability of protein-phenotype associations. Consequently, our MR analysis was broadened to encompass all pQTLs (cis + trans) (**Supplementary Table 3**). In these analyses, a total of 117 protein-feature associations demonstrated MR evidence, including 44 distinct proteins, the majority of which remained undetected in the cis-pQTL analysis (**Figure 3**, **Table 3**, **Supplementary Tables 7**, **8**). Within the newly identified proteins across all pQTLs, STOM, FASLG, ADH1B, IRF9, CYB5R1, TMPRSS11D, TENC1, PRDM1, and KLRF1 stood out as positive proteins that intersect in the IBD, CD, and UC phenotypes.

**Table 3:** cis + trans pQTL-Based MR, Colocalization, and Steiger Filter Analysis: Final Protein Selections Associated with IBD and Its Subtypes.

Protein	Author	Outcome	Direction
A2ML1	Egil Ferkingstad , et al	CD	Positive
ADH1B	Benjamin B. Sun, et al	IBD,CD,UC	Negative
APOBR	Benjamin B. Sun2, et al	IBD	Negative
ARL2	Egil Ferkingstad , et al	CD	Negative
CCL19	Egil Ferkingstad , et al	CD	Negative
CXCL13	Egil Ferkingstad , et al	CD	Negative
ERAP2	Alexander Gudjonsson, et al,Benjamin B. Sun, et al	IBD,CD	Positive
FASLG	Alexander Gudjonsson, et al	IBD,CD,UC	Positive
FCGR2A	Egil Ferkingstad , et al	IBD,UC	Negative
FUT3	Alexander Gudjonsson, et al	CD	Negative
IDO1	Egil Ferkingstad , et al	UC	Negative
IL18R1	Benjamin B. Sun, et al	IBD,CD	Positive
IL1RL1	Benjamin B. Sun, et al	IBD	Positive
IL23R	Karsten Suhre, et al,Benjamin B. Sun, et al,Egil Ferkingstad , et al,Alexander Gudjonsson, et al	IBD,CD,UC	Positive
LHCGR	Egil Ferkingstad , et al	IBD	Positive
MST1	Karsten Suhre, et al,Egil Ferkingstad , et al,Benjamin B. Sun2, et al	IBD,CD,UC	Negative
NEU1	Egil Ferkingstad , et al	CD	Positive
NLGN2	Egil Ferkingstad , et al	IBD,UC	Negative
PARK7	Benjamin B. Sun2, et al	IBD	Positive
PITPNA	Egil Ferkingstad , et al	CD	Negative
PRDM1	Benjamin B. Sun, et al	IBD,CD,UC	Negative
RBP5	Egil Ferkingstad , et al	UC	Positive
STAT3	Egil Ferkingstad , et al	CD	Positive
STOM	Egil Ferkingstad , et al,Benjamin B. Sun, et al	IBD,CD,UC	Negative
TBCA	Egil Ferkingstad , et al	CD	Negative
TENC1	Egil Ferkingstad , et al	IBD,CD,UC	Negative
TMPRSS11D	Egil Ferkingstad , et al	IBD,CD,UC	Negative

Sensitivity analyses confirmed the consistency of all significant associations detected and added 33 associations with MR evidence, encompassing 22 distinct proteins (**Supplementary Tables 9**, **10**).

### 3.3 Co-localization of pQTLs with IBD risk loci

A colocalization analysis was performed to reduce the possible misleading effects of LD on MR- prioritized associations, aiming to determine the likelihood that genetic connections with proteins and phenotypes originated from identical causal variants. Colocalization was performed for proteins with singular instrumental variables and accessible summary-level GWAS data. Among the associations prioritized by cis-MR, 83 were subjected to colocalization analysis, and 40 showed significant signs of colocalization (**Supplementary Table 11**). Furthermore, among the associations identified by MR using all pQTLs, 78 out of 117 analyzed associations demonstrated substantial evidence of colocalization (**Supplementary Table 12**).

### 3.4 Testing causal direction through Steiger filtering analysis

Recognizing the potential of inverse causality in the anticipated associations between proteins and phenotypes, a directional examination, namely the Steiger filtering analysis, was performed to verify if the associations identified by MR truly evolved from proteins to IBD-related traits. The results indicated that the majority of associations identified by MR, including those identified via sensitivity analyses, mainly conformed to the accurate causal direction from proteins to IBD-related traits (**Supplementary Tables 13**-**15**).

### 3.5 Single-cell analysis

**Figure 4:**
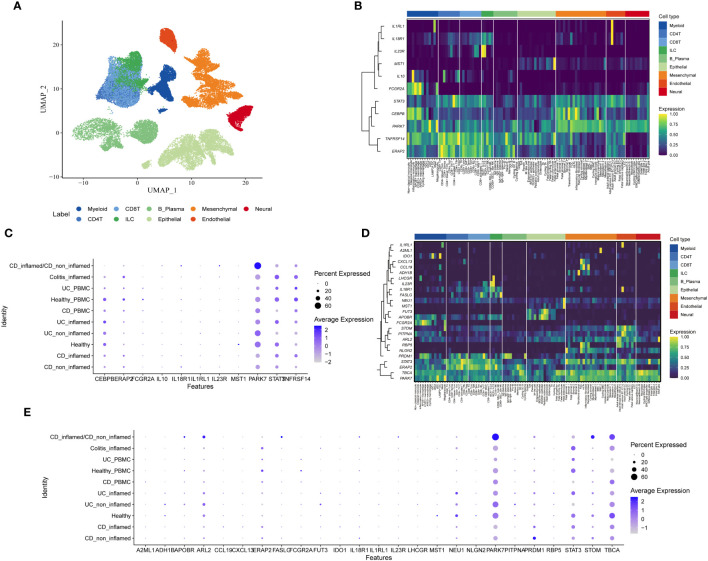
Single-cell type expression of protein-coding genes identified by whole proteome MR in colon tissues. **(A)** Umap showing 9 cell types. **(B)** Heatmap of expression distribution of Cis-Only MR positive proteins in different cell types. **(C)** Bubble chart plot of expression distribution of Cis-Only MR positive proteins in different disease states. **(D)** Heatmap of the expression distribution of cis + trans MR positive proteins in different cell types. **(E)** Bubble chart plot of expression distribution of cis + trans MR positive proteins in different disease states.

The scIBD encompasses 9 primary subtypes (myeloid, CD4 T cells, CD8 T cells, Innate Lymphoid Cells(ILC), B/plasma cells, epithelial cells, mesenchymal cells, endothelial cells, and neuronal cells) and 101 cell subtypes (**Figure 4A**). To understand the specificity of genetically predicted circulating proteins identified by MR analysis across different cell types and disease states, Gene Enrichment Analysis was employed, illustrating the variability among cell subtypes as shown in **Figures 4B, D**. It was observed that among the Cis-Only MR positive proteins, STAT3, CEBPB, PARK7, TNFRSF14, and ERAP2 were expressed at significantly higher levels compared to other proteins across all cell types. Other proteins demonstrated cell-specific expression, such as IL1RL1 and IL18R1 in endothelial cells, IL23R in Innate Lymphoid Cells (ILC), MST1 in epithelial cells, and IL10 and FCGR2A in myeloid cells (**Figure 4B**). Within the cis + trans MR positive proteins, IL1RL1 was highly expressed in endothelial cells, A2ML1 in mesenchymal cells, IDO1 in myeloid cells, and CXCL13 and CCL19 were co-expressed in mesenchymal cells (**Figure 4D**). Furthermore, the differential expression of these proteins across various health and disease states was investigated, revealing that PARK7 was highly expressed in both CD inflamed and CD non-inflamed conditions (**Figures 4C, E**).

### 3.6 Protein-protein interaction network analysis

To deepen our comprehension of the relationship between MR-identified proteins and their enriched functions, thereby deepening our insight into the pathogenesis of IBD and its subtypes, PPI and pathway analyses were undertaken. By employing MR-prioritized proteins identified through cis-pQTLs, the PPI network consisted 14 nodes and formed 14 edges (with an expected number of edges being 3), guided by an interaction score threshold of 0.4 (medium confidence). This result indicates that this network has a notably higher number of interactions than a similarly sized collection drawn at random from the genome (enrichment p-value: 5.73×10^-7) (**Supplementary Figure 1A**). Regarding the proteins identified through cis and trans MR, the PPI network consisted 27 nodes and formed 14 edges, significantly surpassing the expected 4 edges (enrichment p-value: 5.15×10^-5) (**Supplementary Figure 1B**).

### 3.7 Functional enrichment analysis results

The GO pathway enrichment analysis revealed a multitude of biological pathways that were notably enriched and had a strong correlation with IBD. These pathways, enriched among the cis-MR prioritized proteins (**Supplementary Table 17**, **Supplementary Figure 2A**), encompass the generation of interferon-gamma, positive regulation of cytokine production, regulation of adaptive immune responses, modulation of interferon-gamma production, and regulation of Type 1 T helper cell immune responses. The results of this enrichment analysis highlight the critical role of immune regulatory mechanisms in IBD, particularly in the regulation of cytokine production and immune responses. Similar enrichments were observed among the proteins prioritized by cis + trans MR (**Supplementary Table 18**, **Supplementary Figure 2B**).

The KEGG pathway enrichment analysis also revealed significant pathway enrichments related to IBD. Among the cis-MR prioritized proteins, enriched pathways (**Supplementary Table 19**, **Supplementary Figure 2C**) include cytokine-cytokine receptor interaction, IBD, JAK-STAT signaling pathway, Leishmaniasis, and Tuberculosis. These enriched pathways further emphasize the central role of cytokine-mediated cellular signaling in the pathogenesis of IBD, while also revealing potential connections with other inflammatory and immune-mediated diseases. Enrichments were also found among proteins prioritized by cis + trans MR (**Supplementary Table 20**, **Supplementary Figure 2D**).

The results of these enrichment analysis provide new insights into comprehending the pathological processes of IBD, particularly regarding immune responses and cellular signaling. These insights contribute to revealing the intricate network of the development of IBD and present prospective focal points for upcoming treatment approaches.

### 3.8 Evaluating drug targets for MR-preferred proteins

Given the critical role of human proteins as therapeutic targets, we assessed whether proteins supported by MR evidence could be considered drug targets or have druggable properties. Through a search in the DGIdb database, we identified 15 out of 33 proteins that are considered druggable targets, covering a wide range of potential therapeutic strategies. In particular, we reassessed the potential role of these proteins in inflammatory bowel disease (IBD) therapy and immunomodulation. We found, for example, that IDO1 and IL10 genes interact with a variety of drugs, suggesting their importance in regulating immune responses and inflammatory processes. For example, the IDO1 gene demonstrated significant interactions with a variety of unapproved drugs, such as an interaction score of 8.426 with CHEMBL: CHEMBL1224312, highlighting the potential importance of IDO1 in drug development, particularly in the areas of immune regulation and anti-inflammatory therapy. Similarly, the interaction of the IL10 gene with a variety of drugs, including sirolimus, which is approved for the treatment of other diseases, as well as the unapproved PEGILODECAKIN and ILODECAKIN, further emphasizes the critical role of IL10 in the regulation of immune responses and inflammatory processes. These findings not only shed light on the underlying mechanisms of gene-drug interactions, but also provide valuable insights for future drug development and therapeutic strategies, especially in the field of IBD treatment and immunomodulation (**Supplementary Table 21**).

## 4 Discussion

The cause of Inflammatory Bowel Disease (IBD), which includes Crohn’s Disease (CD) and Ulcerative Colitis (UC), continues to be a mystery, with its origins not completely understood. There is a broad consensus that the pathogenesis of this condition is influenced by a multifaceted mix of genetic factors, immune system imbalance, environmental elements, and changes in the gut microbiome (26). Irregular immune reactions are crucial in the emergence and advancement of IBD, marked by an overabundance of immune cells and inflammatory agents, resulting in damage to intestinal tissues, imbalance, and prolonged inflammation (27, 28).

Circulating plasma proteins, including immunoglobulins (antibodies), complement proteins, and other immune modulators, play critical roles in the immune system. They are essential for maintaining immune homeostasis and modulating inflammatory responses (29). Abnormal levels or functionality of these plasma proteins can lead to immune dysregulation and exacerbate inflammatory conditions (30). Leveraging pQTL data from nine different cohorts provided by Zhang et al., supplemented with the latest pQTL data, we constructed MR tools using 10 proteomic GWAS. Sensitivity analyses were conducted on protein-trait associations supported by MR evidence, further validated through Steiger filtering and Bayesian colocalization analyses. Fourteen cis proteins were identified, including CEBPB, ERAP2, FCGR2A, HGFAC, IL10, IL12B, IL18R1, IL1RL1, IL23R, MST1, PARK7, STAT3, TNFRSF14, and TNFRSF6B. Single-cell expression analyses were undertaken to elucidate the specificity of genetically predicted circulating proteins across different cell types. PPI and functional enrichment analyses were performed using the STRING database. GO and KEGG pathway enrichment analyses performed with the ‘ClusterProfiler’ R package revealed key immunoregulatory and cellular signaling pathways associated with IBD. GO analyses highlighted the importance of the regulation of interferon-gamma production and cytokine production, while KEGG analyses highlighted the role of cytokine-cytokine receptor interactions and the JAK-STAT signaling pathway in the central role in the pathogenesis of IBD. These findings reveal a complex network of immune responses and cell signaling regulation in the development of IBD, providing new perspectives for future therapeutic strategies. Finally, the druggability of identified proteins was evaluated in the DGIdb database, along with exploring development information related to potential drugs. This study not only unveiled potential associations between circulating proteins and IBD subtypes but also provided valuable insights for future therapeutic target discovery and drug development through comprehensive bioinformatic analyses.

CEBPB, located downstream of IBD risk loci, is overexpressed in UC mucosa and strategically positioned at the interface of bone marrow and epithelial inflammatory clusters, suggesting its involvement in IBD pathogenesis (31–33). Research by Sudhakar et al. identified CEBPB’s role in CD4+ and CD14+ cells of CD patients (34), while Betal drugs modulate CRP responses to IL-1 via CEBPB (35). Dysregulation of transcription factors, including CEBPB, underscores its potential role in IBD, highlighted by whole-blood transcriptome analyses showing its significant upregulation in IBD (36). Furthermore, CEBPB affects IBD progression by regulating the expression of SCFAs, highlighting the central role of CEBPB in IBD and its potential as a therapeutic target (37).. Tamura et al. demonstrated CEBPB’s key role in monocyte/macrophage regulation, potentially affecting IBD immune responses by upregulating Csf1r expression (38), indicating CEBPB’s core function in IBD pathology and its potential as a therapeutic target.

ERAP2 gene polymorphisms, linked to resistance against lethal infections and susceptibility to autoimmune diseases including IBD (39–41), affect T-cell immune responses by processing antigen peptides (42). The involvement of ERAP2 in immune responses and inflammatory regulation may relate to its role in IBD pathogenesis. Further research is needed to understand the mechanisms linking ERAP2 with IBD and explore the potential of ERAP2 inhibitors as therapeutic interventions.

FCGR2A gene, associated with risks of various autoimmune diseases (43), has a protective effect against UC and CD through its functional variant FCGR2A*519G. Studies by Tomas Castro-Dopico and Kouichi Asano revealed the gene’s significant association with UC susceptibility and its pronounced effect in females (44–46), emphasizing FCGR2A’s importance in IBD pathogenesis and its therapeutic potential.

HGFAC, involved in cell proliferation, migration, and inflammation through activating HGF and MSP, plays roles in metabolic regulation and inflammatory responses (47–49). Its involvement in tissue repair and plasma activation post-injury suggests a role in IBD pathology, potentially influencing intestinal healing in IBD patients, making HGFAC a focus for new therapeutic approaches in IBD management.

IL-10, a key anti-inflammatory cytokine, is crucial for intestinal mucosal homeostasis. Deficiencies in IL-10 or its receptors lead to severe intestinal inflammation in mice and humans (50, 51). IL-10 polymorphism rs3024505 is associated with IBD, particularly in Europeans, highlighting IL-10’s role in UC pathophysiology and its importance in regulating mucosal immune homeostasis (52, 53).

IL12B gene, encoding the shared subunit IL-12p40 of IL-12 and IL-23, is key in IBD pathogenesis by affecting Th1 and Th17 cell differentiation and activation (54). Genetic variations in IL12B are linked to increased IBD susceptibility and disease severity (55, 56) with research indicating the potential of IL12/23p40-targeted immunotherapy for diagnosis and management, reducing invasive procedures like colonoscopy.

IL-23R, the receptor for IL-23, is implicated in IBD development through its role in Th17 cell differentiation and immune response (57, 58). Polymorphisms in IL-23R are associated with IBD risk, with specific mutations leading to abnormal IL-23R signaling and increased disease susceptibility (59, 60). Therapies targeting IL-23, such as ustekinumab, have shown effectiveness in treating moderate to severe CD and UC, underscoring the importance of ongoing research in understanding this pathway and optimizing treatments (61, 62).

IL18 and its receptor IL18R1 play significant roles in IBD pathogenesis. Genetic studies link IL18R1 to IBD susceptibility, with mutations affecting IL-18 signaling and intestinal inflammation (63, 64). Clinical trials exploring IL-12/IL-23 inhibitors’ effects in IBD treatment, alongside the causal relationship between elevated IL18 levels and IBD susceptibility, suggest repositioning existing drugs targeting IL18 signaling for IBD treatment, highlighting the therapeutic potential of targeting the IL18/IL18R1 pathway (61, 65).

IL-1RL1 (also known as IL-33R or ST2), involved in initiating inflammatory responses through IL-33 binding, is associated with IBD risk through gene polymorphisms in IL-33 and IL1RL1 (66, 67). This highlights the potential of the IL-33/IL1RL1 axis in modulating IBD susceptibility and the shared inflammatory pathways between IBD and respiratory diseases like asthma (68, 69), suggesting personalized treatment options for IBD patients resistant to certain therapies.

MST1, involved in various biological processes including cell proliferation and inflammation, is yet to be clearly defined in IBD. However, gene-gene interaction analysis shows its association with CD through polymorphisms in genes like NOD2 and ATG16L1 (70), providing new directions for understanding MST1’s role in IBD.

PARK7 (DJ-1), initially recognized for its role in neurodegenerative diseases, has been identified in the colonic mucosa of IBD children, suggesting its involvement in intestinal inflammation (71, 72). Further research is needed to clarify PARK7’s mechanisms in the gut and the efficacy of PARK7-based therapies in IBD treatment.

STAT3 plays a critical role in IBD pathogenesis, especially in inflammation and fibrosis processes. Its regulation by IL-6 family factors and the role in cell proliferation and chronic inflammation highlight the JAK/STAT3 pathway’s importance in intestinal immunity and IBD (73, 74), suggesting precise STAT3 modulation as a novel IBD treatment strategy.

TNFRSF14 is a member of the tumor necrosis factor receptor superfamily and plays a crucial role in regulating immune responses. Studies have shown that TNFRSF14 expression by innate immune cells has an important role in preventing intestinal inflammation in a T cell transfer model of colitis (75). In a Tnfsf14(-/-) mouse model, it was observed that these mice developed more severe colitis compared to control mice, indicating the protective role of TNFRSF14 in colitis (76).

TNFRSF6B (DcR3), by competitively binding with FasL, TL1A, or LIGHT, plays a role in regulating apoptosis and immune surveillance, impacting intestinal inflammation and CD development (77, 78). Further research is essential to understand the intricate mechanisms of TNFRSF6B/FasR in gut immune regulation and its potential as a therapeutic target in IBD management.

This study possesses significant strengths and limitations. First, the data integration was extensive, covering pQTL data from nine different cohorts and ten proteomics GWAS data, which enhanced the reliability and breadth of the study. The multiple advanced statistical techniques employed, such as Mendelian randomization analysis, Steiger filtering, co-localization analysis, and protein-protein interaction network analysis, ensured a comprehensive examination of the data and minimized bias. In addition, by using data from European populations, the study reduced potential confounders due to population stratification and improved the accuracy of the identified gene associations. Single-cell expression analysis High-resolution single-cell transcriptome analysis using the scIBD platform provided insight into the specific expression of circulating proteins in different cell types, providing valuable insights into the cellular mechanisms of IBD. However, there are some limitations of the study. First, population specificity limits the generalizability of the results to other populations and requires further studies in diverse populations. Despite the exclusion of polytomous instrumental variables, there may still be a degree of polytomousness that confounds the results of MR analyses. The study relied on publicly available datasets, and data quality and completeness may vary, affecting results. In addition, the cross-sectional design limited time-dynamic analysis to draw conclusions about dynamic changes in protein levels and their impact on disease progression. Taken together, the present study not only adds to existing scientific knowledge but also provides important guidance for future research and clinical practice. Future research should focus on validating findings in independent cohorts and diverse populations, exploring the functional mechanisms of proteins in IBD, and designing clinical trials to test the efficacy and safety of new therapeutic targets to advance the understanding and management of IBD.

## 5 Conclusion

This study, through the integration of multi-cohort pQTL data from Zhang et al. and the latest proteomics GWAS, established a MR analytical framework for circulating proteins, identifying 14 cis proteins associated with IBD, including CEBPB and ERAP2. The reliability of protein-trait associations was corroborated through sensitivity analysis, Steiger filtering, and Bayesian colocalization analysis. Single-cell expression analysis and bioinformatics approaches elucidated the specificity and biological functions of these proteins across different cell types. Furthermore, the druggability of these proteins was explored via the DGIdb database, offering fresh insights into therapeutic target discovery and drug development. This lays a foundational groundwork for future research and the formulation of treatment strategies for IBD.

## Data availability statement

The datasets presented in this study can be found in online repositories. The names of the repository/repositories and accession number(s) can be found in the article/**Supplementary Material**.

## Author contributions

BL: Writing – review & editing, Data curation, Formal analysis, Investigation, Methodology, Software, Writing – original draft. PH: Data curation, Software, Writing – original draft, Formal analysis, Writing – review & editing, Methodology, Validation. HL: Data curation, Formal analysis, Writing – original draft, Writing – review & editing, Investigation, Resources, Validation. XZ: Validation, Writing – review & editing, Project administration, Supervision. AZ: Supervision, Validation, Writing – review & editing, Visualization. YX: Visualization, Writing – review & editing, Project administration. BZ: Writing – review & editing, Resources, Supervision. JZ: Supervision, Writing – review & editing, Conceptualization.
