# Supplementary material for: Evaluating the causal effect of circulating proteome on the risk of inflammatory bowel disease-related traits using Mendelian randomization
Source: Front Immunol. 2024 Jul 31;15:1434369. doi: 10.3389/fimmu.2024.1434369 (PMC11321985; doi:10.3389/fimmu.2024.1434369)
Supplement: Supplementary file 1 [file DataSheet_1.docx]

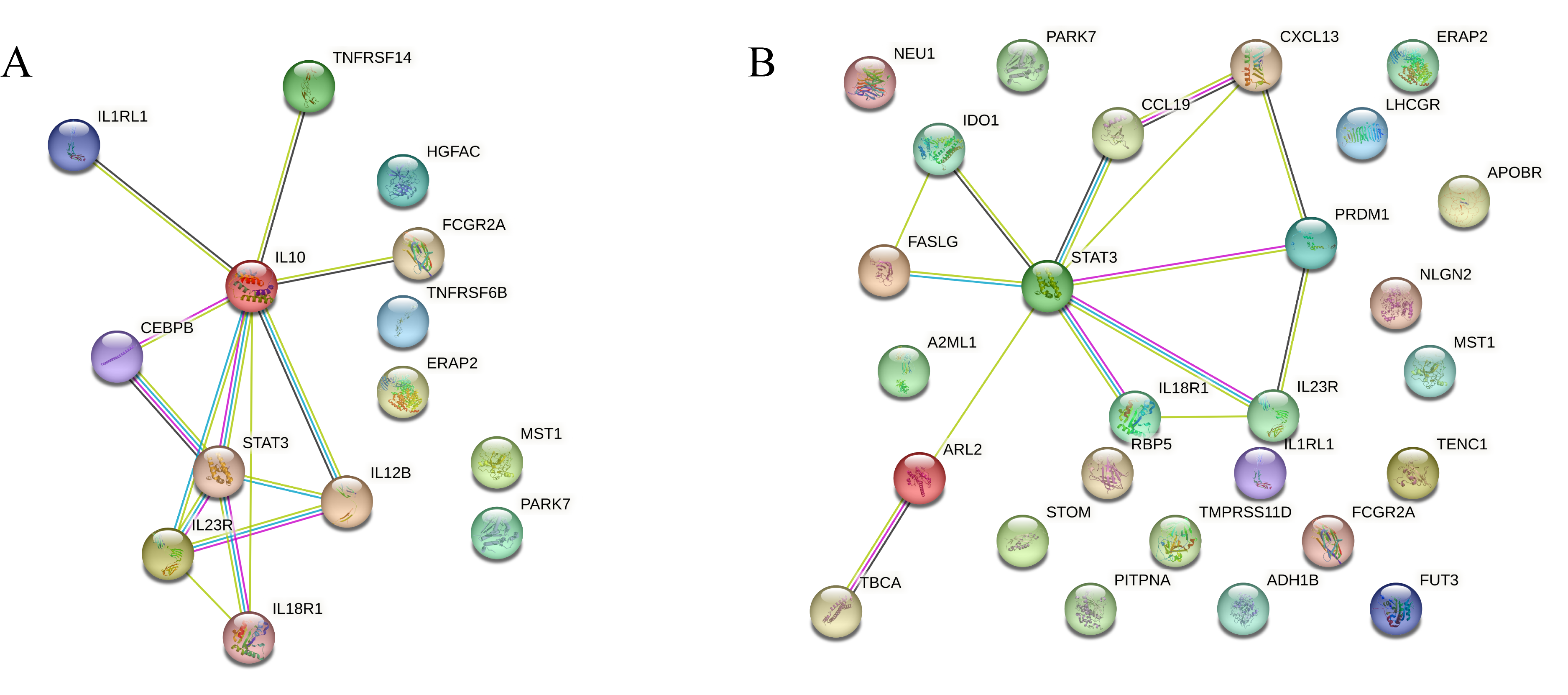


**Supplementary Figure 1**. Protein-protein interaction networks of the MR-prioritized proteins

(A) PPI network of the proteins prioritized by cis-only MR.

(B) PPI network of the proteins prioritized by cis+trans MR.


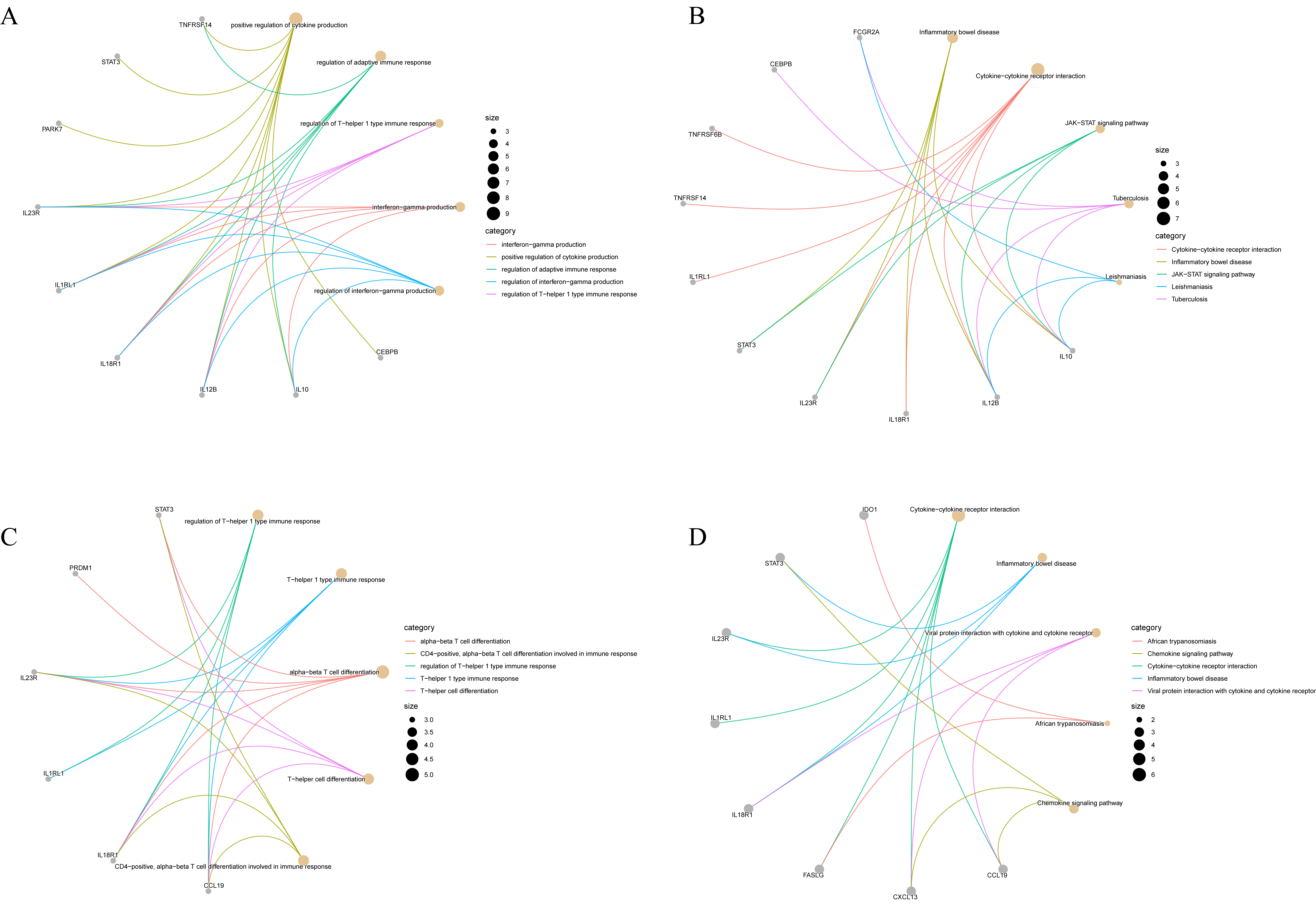


**Supplementary Figure 2**. functional enrichment analysis

(A) go analysis to determine the protein enrichment pathway cis-only MR.

(B) go analysis identifies protein enrichment pathways cis+trans MR.

(C) KEGG determines protein enrichment pathway cis-only MR.

(D) KEGG determines the protein enrichment pathway cis+trans MR.
